# Lived experience narrative: My bipolar odyssey

**DOI:** 10.4102/sajpsychiatry.v30i0.2339

**Published:** 2024-07-19

**Authors:** Samukelisiwe J. Ngcobo

**Affiliations:** 1 Vocal Mentality, Johannesburg, South Africa

I am a doctor. I am also a patient who has been navigating bipolar disorder for twice the lifespan of my medical career. It may seem that I am stating this with natural ease, but it certainly has not been a smooth arrival at this destination because of the stigma attached to my mental illness.

It is my firm belief that the best person to tell the story of a mental illness is the person who is living with it. Sharing this bold thought feels empowering and liberating to me. I hope it will help another person who knows the difficulties that come with a life-journey alongside bipolar disorder. Having bipolar disorder in my life presented various forms of stigma. In a religious context, I was dismissed as ‘demon-possessed’ because of the mania with associated psychosis. From a cultural perspective, I was described as ‘bewitched’ because of the ‘curse of madness’ that had befallen me. In social settings, I was sometimes avoided by others, as though my strange behaviour was perhaps contagious. These stigmatising behaviours from others left me ashamed, isolated and confused, especially during my younger years.

But, the worst form of stigma I have encountered was self-stigma: my own denial of my mental illness. My experience of self-stigma felt like a dark cloud that followed me everywhere. I describe it as the uncommitted sin. Sin is something that is frowned upon and often evokes shame in someone who is deemed guilty of it. Living with mental illness is not a choice that one pursues, hence it is uncommitted.

It was external stigma that taught me to self-stigmatise by denying my mental illness. The lack of insight and associated shame took me along a tumultuous journey of multiple relapses and several admissions to psychiatric facilities.

Unfortunately, I believe that the quality of care that I received was a contributor to my recurrent relapses. I experienced the approach of mental healthcare providers to be paternalistic and punitive. I resented this and the resentment was deepened by the frustration of caregivers and loved ones that I encountered who claimed that I deliberately refused treatment. They failed to probe and interrogate my reasons for non-adherence. A lot of my defiance was because of a lack of understanding. I lacked insight about my diagnosis and this impaired my judgement. I did not understand my illness and therefore could not accept it.

Psycho-education brought an important paradigm shift to my life. I finally met my current psychiatrist who was a perfect fit for me, and remains so. They advocated for my holistic mental well-being. This approach turned my life around positively. My prognosis improved because of their empathetic and comprehensive intervention. One of the greatest gifts that I gained with my psychiatrist’s support was empowerment through self-advocacy. Now I feel as if I am a part of my management team because I have agency and a voice regarding my well-being interventions. I feel that my voice matters and that my insights are embraced and valued. I have the utmost respect for the multidisciplinary team management approach because its application has granted me quality of life.

It took me 16 years to make peace and accept my diagnosis. I can confidently say that I am not suffering from bipolar disorder. I am thriving despite it.

Samukelisiwe J. Ngcobo is a medical doctor, author and international speaker who is a mental health activist and founder of Vocal Mentality. Her personal platforms www.vocalmentality.com and Sisters For Mental Health have a focus of destigmatising mental illness and educating society about mental health.

